# A novel blood collection technique from the rat thoracic aorta: employing a diaphragm sac as a reservoir

**DOI:** 10.1186/s42826-026-00277-7

**Published:** 2026-04-08

**Authors:** Purvaj G. Barote, Abdulla Sherikar, Sameer N. Goyal, Sanjay N. Awathale

**Affiliations:** Department of Pharmacology, School of Pharmacy & Technology Management, SVKM NMIMS Global University, Dhule, Maharashtra 424001 India

**Keywords:** Blood collection, Thoracic aorta, Diaphragm sac

## Abstract

**Background:**

Several blood withdrawal techniques are employed for biochemical research. However, these techniques fail to fulfil the desired requirement of blood volume. We invented the alternative method of blood collection through the thoracic aorta in an anesthetized rat. Under anesthesia, the rat was placed supine, and a transverse abdominal incision was made. The diaphragm was carefully cut, and a vertical thoracic incision was performed. The rat was held upright, creating a diaphragm sac, as a natural reservoir for blood collection. The thoracic aorta was identified and cut, allowing a large volume of blood to accumulate and be collected in sample tubes. The purity of the blood samples was determined using the plasma protein concentration, hemolysis index and lactate dehydrogenase (LDH) activity.

**Results:**

A maximum blood volume of approximately 10–15 mL was successfully collected from the thoracic aorta using the diaphragm sac in a single rat. This method provided a higher blood yield compared to traditional techniques, such as retro-orbital bleeding, tail vein sampling, and cardiac puncture, all of which typically yield smaller volumes and require multiple collections. The blood collected using this method demonstrated comparable quality to that obtained via cardiac puncture, with no significant differences observed in plasma protein concentration, hemolysis index and LDH activity, supporting its suitability for high-quality plasma collection.

**Conclusions:**

Existing blood collection methods often fall short of volume requirements, leading to repeated needle and capillary use and causing animal stress. In contrast, the proposed method is simpler, designed for rats sacrificed post-procedure. The new blood collection method through the thoracic aorta efficiently provides large blood volumes, ideal for biochemical investigations.

## Background

Accurate and efficient blood withdrawal in laboratory rats is crucial for various biomedical research applications. Traditional methods such as retro-orbital bleeding, tail vein sampling, and cardiac puncture have been widely utilized, each presenting distinct advantages and limitations concerning the volume of blood obtained, the welfare of the animal, and the quality of the sample. However, these conventional techniques often fall short when large blood volumes are required, necessitating the exploration of alternative methods. Retro-orbital bleeding, for instance, allows for the collection of moderate blood volumes (0.5–3 mL) but poses risks of ocular injury and requires anesthesia in rats [1]. Saphenous vein sampling is less invasive, can be performed without anesthesia, and permits repeated small-volume draws, making it suitable for pharmacokinetic studies [2]. Tail vein sampling is less invasive and can be performed without anesthesia, yet it typically yields smaller volumes and may require warming of the tail to enhance blood flow [3]. Cardiac puncture enables the collection of 5–10 mL of blood in rats but is strictly a terminal procedure performed under deep anesthesia. It requires considerable technical skill and, if the animal has dextrocardia, the sampling may be unsuccessful [2, 4]. Another commonly used terminal blood collection method involves sampling from an abdominal vessel such as the posterior vena cava. This approach can yield 5–10 mL, making it suitable for terminal studies. However, it generally requires more extensive dissection and multiple (three to four) repetitions, and carries a higher risk of sample contamination or coagulation within the syringe [4]. In contrast, our thoracic aorta method, with the diaphragm sac serving as a reservoir, allows efficient retrieval of large, high-quality samples with relatively straightforward access. While both methods are appropriate for terminal endpoint studies, the thoracic aorta approach may offer improved sample quality and procedural efficiency, whereas abdominal vessel collection can be an alternative depending on experimental constraints. These limitations are particularly critical in modern research applications, such as multi-omics and pharmacokinetic studies, which demand large volumes of high-quality blood to ensure reliable downstream analyses. The comparison of various blood collection techniques in rats is mentioned in Table 1.

**Table 1:** Summary of commonly used blood collection methods in rats, including typical volumes, limitations, and procedural types

Sr. No.	Method	Volume collected (mL)	Limitations	References
**Survival Methods**				
1.	Retro-orbital bleeding	0.5–3	- Requires anesthesia - Risk of ocular injury - Requires trained personnel - Requires repeated sampling	[1, 5]
2.	Tail vein sampling	0.05–0.1	- Requires a proper restraint - May need tail warming - Requires skilled personnel	[3]
3.	Saphenous vein sampling	0.3–1	- Requires proper restraining - Risk of hemolysis - Inconsistent sample quality	[2, 6, 7]
4.	Jugular vein sampling	2–3	- Requires anesthesia - Technically challenging - Risk of infection	[8]
5.	Submandibular bleeding	0.2–1	- Risk of scarring - Requires moderate restraint	[9]
6.	Facial vein sampling	0.2–0.5	- Require anesthesia - Risk of tissue trauma	[10]
7.	Dorsal pedal vein	0.2–0.4	- Risk of hematoma	[11]
**Terminal methods**				
1.	Cardiac puncture	5–10	- Requires deep anesthesia - Risk of contamination - Risk of infection - Requires repeated sampling - Requires skilled personnel	[2, 4]
2.	Posterior vena cava	5–10	- Requires deep anesthesia - Requires extensive dissection - Risk of sample contamination - Potential for coagulation in the syringe - Requires repeated sampling	[4]
3.	Thoracic aorta blood sampling from the diaphragm sac (present study)	10–15	- Requires deep anesthesia	

In light of these limitations, we have developed a novel blood collection technique via the thoracic aorta in anesthetized rats. This method allows for the collection of approximately 10 mL of blood, with volumes reaching up to 15 mL following compression of the abdominal aorta, surpassing the volumes obtainable through traditional methods. By carefully accessing the thoracic aorta through a transverse abdominal incision and utilizing the diaphragm as a natural reservoir, this technique minimizes animal distress and enhances sample quality. Our method passed the purity and quality assessment parameters such as plasma protein concentration, hemolysis index and lactate dehydrogenase (LDH) activity when compared to cardiac puncture technique. Therefore, this approach offers a significant advancement in preclinical laboratory practices requiring substantial blood volumes, providing a more efficient and humane alternative to existing methods.

## Methods

**Fig. 1:**
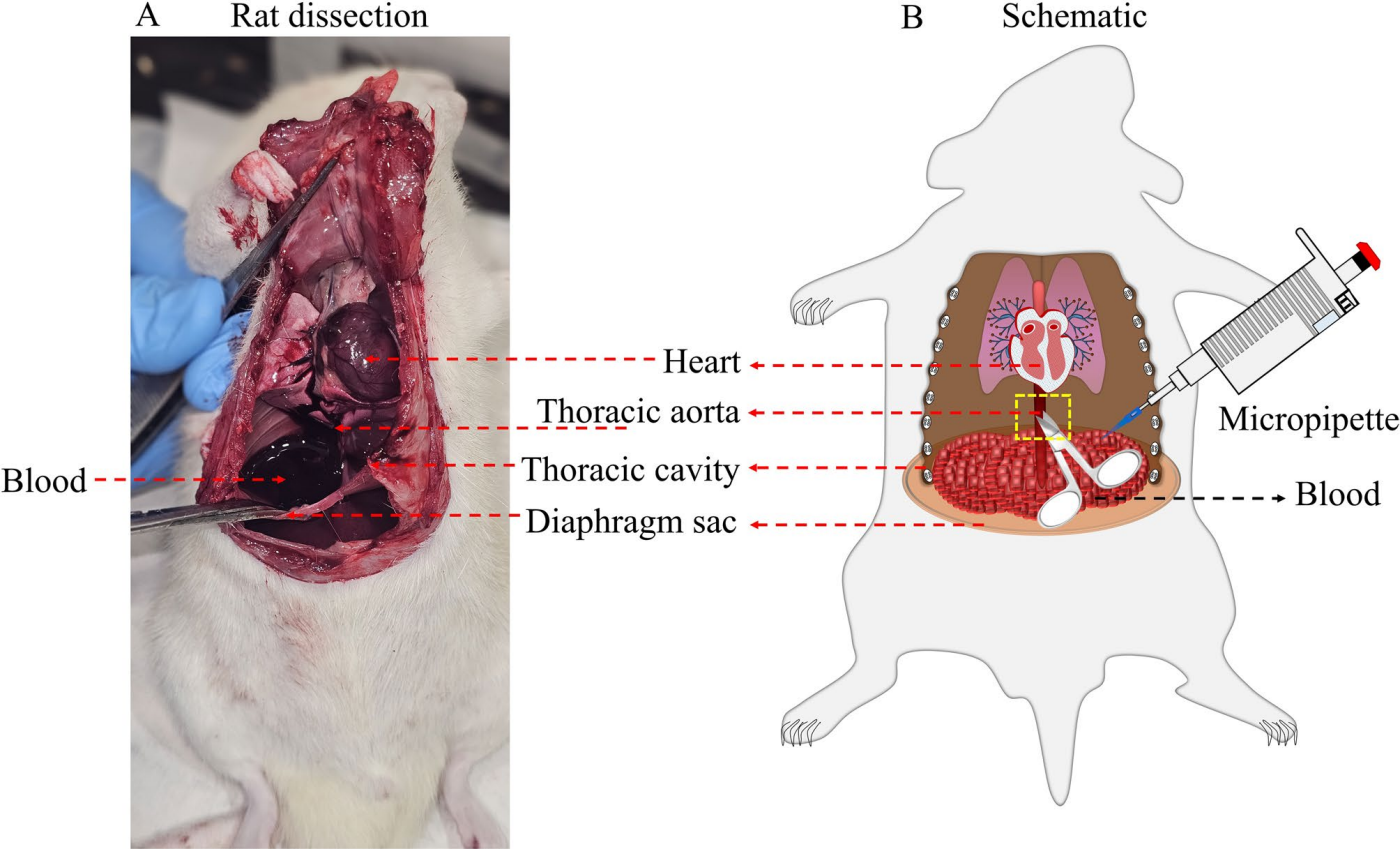
Diagram indicating the location of the thoracic aorta and the collection of blood on the diaphragm sac in a dissected animal (**A**) and the schematic representation (**B**)

**Fig. 2:**
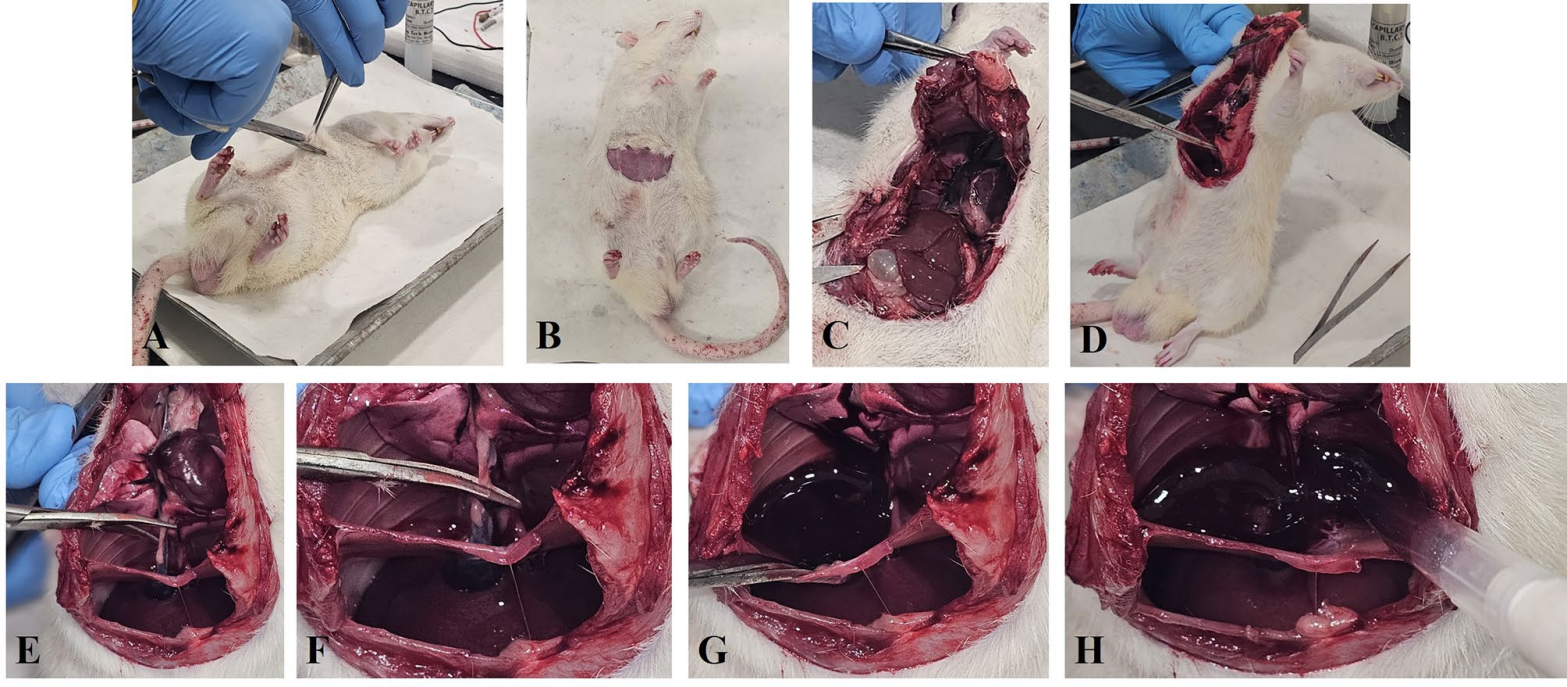
Stepwise illustration of the novel thoracic aorta blood collection technique in anesthetized rats. Anesthesia and positioning (**A**), abdominal incision (**B**), opening of the thoracic cavity (**C**), exposure of diaphragm sac and thoracic cavity (**D**), identification of the thoracic aorta (**E**), lifting the aorta (**F**), and blood collection using a micropipette (**G** and **H**)
- **Anesthesia and positioning** The rats were anesthetized using thiopentone sodium[65 mg/kg, intraperitoneal (ip), Neon Laboratories Ltd., Mumbai, India]. Deep anesthesia is required when performing the thoracic aorta blood collection method to ensure complete analgesia and immobility, minimizing stress and discomfort for the animal. Proper anesthesia also facilitates safe and efficient sample retrieval while maintaining high sample quality. Animals should be carefully monitored throughout the procedure to confirm adequate anesthetic depth. Thereafter, the rat was placed in the supine position on a sterile surgical surface (Fig. 2A).
- **Surgical field preparation and abdominal incision** A 5-8 cm transverse incision was made in the lower abdominal region using sterile surgical scissors and scalpels to carefully expose the underlying abdominal muscles. Following this, a second, deeper incision was made through the abdominal muscle layers to access the peritoneal cavity. Throughout the procedure, care was taken to avoid damaging surrounding tissues and organs (Fig. 2B).
- **Opening of the thoracic cavity and aspiration of surrounding fluids** After the incision of the abdominal muscles, the diaphragm was carefully and partially incised along its central tendon, ensuring that the lower portion remained intact to preserve structural support. This partial incision allowed initial access to the thoracic cavity and clear visualization of the thoracic organs, including the heart, lungs, and major vessels such as the thoracic aorta. To fully open the thoracic cavity, the dissection was then extended laterally on both sides, running parallel to the thoracic cage and continuing upward toward the forelimbs. The ribs and intercostal muscles were gently separated using blunt dissection and fine scissors to minimize tissue trauma and prevent accidental puncture of internal organs. This systematic approach enabled controlled exposure of the thoracic cavity while maintaining anatomical integrity and minimizing contamination risk during subsequent procedures (Fig. 2C).
- **Precise handling of the rat and diaphragm sac** To facilitate blood collection from the thoracic aorta, the dissected rat was gently lifted and held in an upright, vertical position. This posture allowed gravity to pull the partially incised diaphragm downward, causing it to sag and form a natural sac-like cavity beneath the heart. This temporary reservoir helped collect any residual thoracic fluids away from the major vessels and provided a clear, unobstructed view of the thoracic aorta. Maintaining this position also minimized the risk of blood contamination from surrounding tissues and improved accessibility for precise blood withdrawal (Fig. 2D). Fluid such as pleural fluid, tissue fluid, or sometimes abdominal fluid was carefully removed from the diaphragm sac using a sterile pipette to prevent contamination of the blood sample.
- **Cleaning of the diaphragm sac** Any visible pericardial or pleural fluid was gently aspirated using a sterile pipette to prevent contamination of the blood sample.
- **Identification of the thoracic aorta** Hold the rat in the same upright position, and carefully remove the connective tissue surrounding the heart and major blood vessels using fine forceps and scissors. Take care to avoid damaging any structures during this process. The thoracic aorta is located posterior to the heart, running parallel and close to the vertebral column. Once exposed, it can be clearly identified as a large, pulsatile vessel suitable for blood collection (Fig. 2E).
- **Exposure of the thoracic aorta** The heart was gently lifted using blunt forceps to expose the thoracic aorta, located posterior to the heart and running parallel to the vertebral column. The surrounding connective tissue was carefully cleared to isolate the vessel without applying clamps or undue tension (Fig. 2F).
- **Incision of the aorta and blood pooling** Once fully exposed, the thoracic aorta was sharply incised using fine surgical scissors to allow blood to flow freely into the diaphragm sac, which served as a natural reservoir for collection (Fig. 2G). Care was taken to avoid contact with surrounding tissues to minimize contamination and hemolysis.
- **Blood collection using a micropipette** At this point, blood was promptly collected from the diaphragm sac using a micropipette, ensuring the desired volume was drawn carefully to maintain sample integrity.
- **Sample purity and quality assessment** In the present study, *n* = 8 rats were used for blood withdrawal using the thoracic aorta and *n* = 8 rats were used for the cardiac puncture technique. The collected blood samples (Table 2) were divided into two volumes; one was processed for quantification of plasma protein concentration and hemolysis index. The second volume of blood sample was used for the estimation of serum lactate dehydrogenase (LDH) activity.

- **Animals** Adult male Sprague-Dawley rats, ten to twelve weeks old (250-300 g body weight) were used in the present study. Protocols employed in the present study were carried out under strict compliance with the Institutional Animal Ethics Committee (IAEC), SVKMs Institute of Pharmacy, Dhule, Maharashtra, India (approval No. SVKM-IOP/IAEC/2024/December/03).
- **Stepwise procedures for blood withdrawal from the thoracic aorta** The dissection and schematic images represent the exact location of the thoracic aorta and diaphragm sac (Fig. 1). As depicted in Fig. 2, the following are the steps used for the collection of blood from the thoracic aorta. All animals included in the study were maintained under similar conditions, including comparable body weight, health status, and anesthetic depth. This ensured that the physiological state was consistent across groups, allowing meaningful comparison of the volume of blood collected between methods.

**Table 2:** Blood volumes obtained from rats (250–300 g body weight) using cardiac puncture (*n* = 8) and thoracic aorta collection using the diaphragm sac reservoir (*n* = 8)

Collected blood volume (mL) from t**horacic aorta using the diaphragm sac reservoir**		Collected blood volume (mL) from cardiac puncture	
Animal 1	10.4	Animal 1	5.3
Animal 2	14.5	Animal 2	6.2
Animal 3	13.2	Animal 3	7.3
Animal 4	16.1	Animal 4	4.3
Animal 5	15.3	Animal 5	8.8
Animal 6	11.5	Animal 6	5.2
Animal 7	14.3	Animal 7	5.7
Animal 8	12.9	Animal 8	9.5
- **Plasma protein concentration** The blood samples were collected in centrifuge tubes containing 0.1% EDTA were centrifuged at 2000 × g for 10 minutes at 4 °C. The plasma supernatant was carefully collected to avoid contamination with cellular components. Plasma protein concentration was determined using the bicinchoninic acid (BCA) assay and bovine serum albumin (BSA) as a standard. Plasma samples and standard (10 µl) were loaded in the 96-well plate and mixed with BCA reagent (A and B, 50:1, respectively). The mixture was incubated at 37 °C for 30 minutes, and the absorbance was measured at 562 nm using a microplate reader (BioTek Synergy H1, Microplate reader). The protein concentration of each sample was calculated from the standard curve and expressed as mg/mL [12–15]. All samples were analyzed in triplicate.
- **Hemolysis index** The calculation of the hemolysis index in rat plasma serves as a quality control measure to assess the integrity of the blood samples and to detect contamination by intracellular components, particularly hemoglobin released from lysed red blood cells [16]. The hemolysis index was determined manually by spectrophotometric measurement of free hemoglobin in plasma samples. A total volume of 100 µL of each plasma sample and hemoglobin standards (0-200 mg/dL; Haemoglobin from human blood, Cat. No. H7379, Sigma-Aldrich) was loaded into a 96-well microplate in triplicate. The absorbance was measured at 414 nm using a microplate reader. A standard curve was generated by plotting absorbance values against known haemoglobin concentrations, and the free haemoglobin concentration in each plasma sample was interpolated from the curve and expressed in mg/dL. Plasma samples were classified as non-hemolyzed if the free haemoglobin concentration was below 20 mg/dL [17].
- **Lactate dehydrogenase (LDH) activity** This assay provides a reliable way to quantify LDH activity as an indicator of tissue damage in rat serum [18]. After the successive blood withdrawal into the Eppendorf tubes, allowed to clot at room temperature for 30 to 60 minutes. Thereafter, these sample tubes were centrifuged at 1,500 to 2,000 × g for 10 minutes at 4 °C to separate the serum. Assay reagents consisted of 100 mM Tris-HCl buffer (pH 7.4), 2.5 mM nicotinamide adenine dinucleotide (NAD^+^; Cat. No. 53-84-9, Sigma Aldrich), 50 mM sodium L-lactate as the substrate (Cat. No. L7022, Sigma Aldrich), and 0.5 mM WST-8 (Cat. No. 193,149-74-5, Sigma Aldrich). Serum samples (10 µL) were loaded into a 96-well plate in triplicate. Subsequently, 50 µL of Tris-HCl buffer, 10 µL NAD^+^, 10 µL lactate substrate, and 20 µL WST-8 reagent were added to each well, mixed gently, and incubated at 37 °C for 30 minutes. Absorbance was then measured at 450 nm using a microplate reader. For the standard curve, 10 µL of purified L-Lactate dehydrogenase enzyme (Cat. No. 9001-60-9, Sigma Aldrich) was loaded in triplicate wells and processed identically with the assay reagents. The enzyme activity was calculated and expressed in U/mL.
- **Blood collection using cardiac puncture** For cardiac puncture blood collection, another group of rats (*n* = 8) was anesthetized with thiopentone sodium (65 mg/kg, i.p.) and positioned supine with the chest area exposed. The skin over the sternum was disinfected using an appropriate antiseptic. A sterile 5 mL syringe fitted with a 23 G needle was used to perform the puncture. The needle was inserted at a 45° angle through the chest wall into the left ventricle, and up to 5-10 mL of blood was slowly withdrawn [2]. The collected blood was then processed for evaluation of purity and quality parameters as described above.
- **Statistical analysis** The GraphPad Prism v8.4.2 software was used for the data analysis. The normal distribution of data was calculated by using the online calculator present at the Statistics Kingdom website (https://www.statskingdom.com/shapiro-wilk-test-calculator.html); all the data were found to be normally distributed. Grubb’s test revealed that no outlier was detected in the data. All the data were represented as mean ± standard error of mean (SEM) and analysed using an unpaired t-test. Differences were considered significant if *p* < 0.05.

## Results

**Fig. 3:**
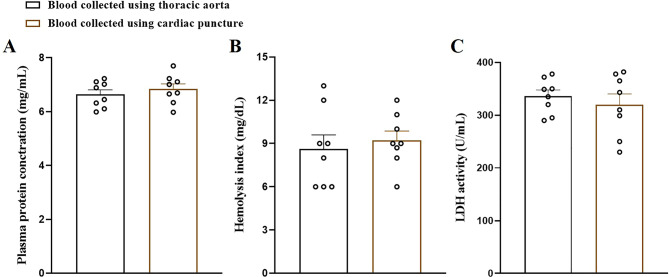
The plasma protein concentration (**A**), hemolysis index (**B**) and serum lactate dehydrogenase (LDH) activity (**C**) were estimated using blood samples collected from the thoracic aorta and cardiac puncture techniques (*n* = 8 rats/group). The data were analysed by an unpaired t-test and expressed as mean ± SEM

- **Large blood volume collected using the thoracic aorta**
  The data showed that approximately 10 mL of blood was collected immediately after cutting the thoracic aorta in each rat (*n* = 8). Additional volume was obtained by gently pressing the abdominal aorta below the diaphragm, which filled the diaphragm sac with residual blood (Fig. 2H), increasing the total yield to 13–15 mL per animal. Together, this method enabled efficient collection of large blood volumes in a single terminal procedure.

- **Plasma protein concentration, hemolysis index and LDH activity in blood samples collected from the thoracic aorta and cardiac puncture**
  To assess the purity and quality of blood samples collected from the thoracic aorta via the diaphragm sac, plasma protein concentration (*t* = 0.7880, df = 14; Fig. 3A), hemolysis index (*t* = 0.5056, df = 14; Fig. 3B), and LDH activity (*t* = 0.7038, df = 14; Fig. 3C) were measured and compared with those obtained via the standard cardiac puncture method. An unpaired t-test revealed no significant differences in plasma protein concentration, hemolysis index, or LDH activity between the two methods (all *p* > 0.05; Fig. 3), indicating comparable sample quality.

## Discussion

Efficient and reliable blood collection in rodents is a fundamental yet often challenging aspect of preclinical research, with implications for both data quality and animal welfare [19]. Standard methods such as tail vein puncture, retro-orbital bleeding, cardiac puncture, and saphenous vein sampling each present specific limitations [20]. Tail vein sampling often requires pre-warming of the animal to dilate the vessels and facilitate blood flow. Despite being minimally invasive, it typically yields small volumes of blood, particularly during repeated or serial collections, and may cause stress due to prolonged handling. Retro-orbital bleeding, while efficient in obtaining relatively larger volumes, is highly invasive and associated with significant animal welfare concerns, including the risk of ocular damage and pain, leading to restrictions or bans in many institutions. Cardiac puncture allows for the collection of large, high-quality blood volumes, making it ideal for terminal procedures. However, it requires a high level of technical skill and anatomical knowledge to perform correctly. Improper technique can lead to failed puncture, internal injury, or blockage of the needle due to contact with cardiac tissue or clot formation. Furthermore, because it is a terminal method, it is unsuitable for longitudinal or survival studies. Although cardiac puncture carries a potential risk of sample contamination, the thoracic aorta method used in this study minimized this risk through direct vessel access under aseptic conditions. In our experiments, blood was collected immediately from the diaphragm sac, which served as a clean reservoir and reduced external exposure. Therefore, even though the dissection was slightly more extensive, the risk of sample contamination was not increased compared to cardiac puncture. Since our procedure was terminal, the risk of infection to the animal was not a concern. Saphenous vein sampling, although less invasive and suitable for repeated collections, is technically demanding and often results in variable blood yield and inconsistent sample quality, especially in inexperienced hands [4, 21]. These limitations underscore the need for alternative strategies that can reliably collect large volumes while minimizing operator variability and animal usage.

In this study, we present a blood collection method for terminal endpoint studies requiring large-volume, high-quality samples, utilizing the thoracic aorta and the diaphragm sac as a reservoir to enable efficient sample retrieval. This technique consistently yielded approximately 10 mL of blood per rat, and volumes of up to 15 mL were obtained by gently pressing the abdominal aorta. This yield is substantially greater than the volumes typically obtained through non-terminal methods such as tail vein, saphenous vein, or retro-orbital sampling, which generally allow for only 0.2 to 1.5 mL of blood per collection, depending on the technique used and sampling frequency [2]. Importantly, as this approach is terminal, it eliminates cumulative stress and potential complications associated with repeated blood sampling in survival studies and aligns with the principles of the 3Rs by reducing animal distress in protocols where repeated sampling is not feasible or ethically acceptable.

However, this technique shares several limitations common to other terminal methods. Notably, it requires deep anesthesia, similar to cardiac puncture, to ensure the procedure is performed humanely and without distress. Furthermore, as this method is terminal, it is not suitable for longitudinal or survival studies. Although the procedure involves opening the abdominal and thoracic cavities, it is performed post-anesthesia and immediately prior to euthanasia, making it ethically acceptable under approved protocols. While opening the thoracic cavity is technically classified as invasive, in the context of a terminal procedure, it does not increase animal suffering when performed under proper anesthesia. Animal distress is minimized through the use of adequate anesthetic depth and rapid handling to reduce procedural time.

In terms of advantages, present method allows for direct access to a large blood vessel, enabling the collection of greater volumes of blood with minimal contamination and low hemolysis, as supported by the plasma protein concentration, hemolysis index, and LDH activity data (Fig. 3). Unlike cardiac puncture, which may risk needle blockage due to contact with myocardial tissue or clot formation, the blood accumulated on the diaphragm sac provides a clear and unobstructed path for blood withdrawal, improving success rates and consistency across samples. Additionally, this approach avoids manipulation of smaller or more fragile veins, such as the tail or saphenous vein, which often yield low volumes and require significant technical skill. Therefore, while this method is limited to terminal studies, it presents a reliable and efficient alternative for obtaining high-quality blood samples with reduced technical variability.

In summary, while this terminal method is not intended to replace existing blood collection techniques, it is most appropriate for terminal endpoint studies that require large-volume, high-quality samples, rather than for survival or longitudinal designs. Its reproducibility and efficiency can help to reduce overall animal usage while supporting reliable biochemical analyses and enhancing the quality of translational research outcomes.

## Conclusions

Blood collection in rats is often limited by existing techniques. The thoracic aorta method via the diaphragm sac offers a simple, reproducible terminal approach that yields larger blood volumes, making it valuable for studies requiring substantial blood samples. Although not suitable for longitudinal studies, this approach enhances sampling efficiency, can reduce animal use, and serves as a practical complement to current methods in preclinical research, making it most applicable to terminal endpoint studies requiring substantial, high-quality samples instead of survival or longitudinal designs.

## Data Availability

The corresponding author, Sanjay N. Awathale, will make all the data behind the study’s conclusions available to the public under reasonable request.

## References

[1] Sharma A, Fish BL, Moulder JE, Medhora M, Baker JE, Mader M, et al. Safety and blood sample volume and quality of a refined retro-orbital bleeding technique in rats using a lateral approach. Lab Anim (NY). 2014;43:63–66. 10.1038/laban.432. 24451361 10.1038/laban.432 PMC3989930

[2] Beeton C, Garcia A, Chandy KG. Drawing blood from rats through the saphenous vein and by cardiac puncture. J Vis Exp. 2007;7:266. 10.3791/266. 10.3791/266 PMC2565848 18989437

[3] Lee G, Goosens KA. Sampling blood from the lateral tail vein of the rat. J Vis Exp. 2015;99:e52766. 10.3791/52766. 10.3791/52766 PMC4542852 26065632

[4] Parasuraman S, Raveendran R, Kesavan R. Blood sample collection in small laboratory animals. J Pharmacol Pharmacother. 2010;1:87–93. 10.4103/0976-500X.72350. 21350616 10.4103/0976-500X.72350 PMC3043327

[5] Van Herck H, Baumans V, Brandt CJ, Boere HA, Hesp AP, van Lith HA, et al. Blood sampling from the retro-orbital plexus, the saphenous vein and the tail vein in rats: comparative effects on selected behavioural and blood variables. Lab Anim. 2001;35:131–39. 10.1258/0023677011911499. 11315161 10.1258/0023677011911499

[6] McClure DE. Clinical pathology and sample collection in the laboratory rodent. Vet Clin N Am Exot Anim Pract. 1999;2:565–90, vi. 10.1016/s1094-9194(17)30111-1. 10.1016/S1094-9194(17)30111-1 PMC7110626 11229044

[7] Patel NJ, Wickremsinhe E, Hui Y-H, Barr A, Masterson N, Ruterbories K, et al. Evaluation and Optimization of blood micro-sampling methods: serial sampling in a cross-over design from an individual mouse. J Pharm Pharm Sci. 2016;19:496–510. 10.18433/J3NK60. 28057168 10.18433/J3NK60

[8] Thrivikraman KV, Huot RL, Plotsky PM. Jugular vein catheterization for repeated blood sampling in the unrestrained conscious rat. Brain Res Brain Res Protoc. 2002;10:84–94. 10.1016/s1385-299x(02)00185-x. 12431707 10.1016/s1385-299x(02)00185-x

[9] Hoggatt J, Hoggatt AF, Tate TA, Fortman J, Pelus LM. Bleeding the laboratory mouse: not all methods are equal. Exp Hematol. 2016;44:132–7.e1. 10.1016/j.exphem.2015.10.008. 26644183 10.1016/j.exphem.2015.10.008 PMC5810935

[10] Harikrishnan VS, Hansen AK, Abelson KS, Sørensen DB. A comparison of various methods of blood sampling in mice and rats: effects on animal welfare. Lab Anim. 2018;52:253–64. 10.1177/0023677217741332. 29165033 10.1177/0023677217741332

[11] Kumar M, Dandapat S, Sinha M, Kumar A, Raipat B. Different blood collection methods from rats: a review. Balneo Res J. 2017;8:46–50. 10.12680/balneo.2017.141.

[12] Awathale SN, Shirasath KR, More BR, Pardeshi GN, Goyal SN, Nakhate KT. Naringenin prevents ethanol-induced reward behavior in adolescent rats through inhibition of TRPM3-dependent inflammation in the pVTA. J Nutr Biochem. 2025;146:110078. 10.1016/j.jnutbio.2025:146:110078. 40825444 10.1016/j.jnutbio.2025.110078

[13] Pardeshi GN, Ali N, Shirasath KR, Goyal SN, Nakhate KT, Awathale SN. Inhibition of TRPM3 channels in the medial prefrontal cortex mitigates OCD symptoms following traumatic brain injury. Inflammopharmacology. 2025;33(5):2849–68. 10.1007/s10787-025-01763-5. 40372651 10.1007/s10787-025-01763-5

[14] Chaudhari AK, Shirasath KR, Goyal SN, Nakhate KT, Awathale SN. A novel biting rod model for assessing aggressive behavior in restraint-stressed rats: role of 5-HT3 receptor and NF-κB-kynurenine pathways. Physiol Behav. 2025;302:115091. 10.1016/j.physbeh.2025.115091. 40912348 10.1016/j.physbeh.2025.115091

[15] Shirasath KR, Chaudhari AK, Nakhate KT, Goyal SN, Awathale SN. Plumbagin alleviates mild traumatic Brain injury-induced obsessive-compulsive disorder in mice by inhibiting nNOS and augmenting Cortico-striatal serotonin levels. Phytother Res. 2025;39(11):5282–301. 10.1002/ptr.70105. 41057257 10.1002/ptr.70105

[16] Sæbø IP, Bjørås M, Franzyk H, Helgesen E, Booth JA. Optimization of the hemolysis assay for the assessment of cytotoxicity. Int J Mol Sci. 2023;24:2914. 10.3390/ijms24032914. 36769243 10.3390/ijms24032914 PMC9917735

[17] Brunori P, Masi P, Faggiani L, Villani L, Tronchin M, Galli C, et al. Evaluation of bilirubin concentration in hemolysed samples, is it really impossible? The altitude-curve cartography approach to interfered assays. Clin Chim Acta. 2011;412:774–77. 10.1016/j.cca.2011.01.010. 21238446 10.1016/j.cca.2011.01.010

[18] Klein R, Nagy O, Tóthová C, Chovanová F. Clinical and diagnostic significance of lactate dehydrogenase and its isoenzymes in animals. Vet Med Int. 2020;2020:5346483. 10.1155/2020/5346483. 32607139 10.1155/2020/5346483 PMC7313120

[19] Ahrens Kress AP, Zhang Y, Kaiser-Vry AR, Sauer MB. A comparison of blood collection techniques in mice and their effects on welfare. J Am Assoc Lab Anim Sci. 2022;61:287–95. 10.30802/AALAS-JAALAS-21-000129. 35314020 10.30802/AALAS-JAALAS-21-000129 PMC9137285

[20] Diehl KH, Hull R, Morton D, Pfister R, Rabemampianina Y, Smith D, et al. A good practice guide to the administration of substances and removal of blood, including routes and volumes. J Appl Toxicol. 2001;21:15–23. 10.1002/jat.727. 11180276 10.1002/jat.727

[21] Aasland KE, Skjerve E, Smith AJ. Quality of blood samples from the saphenous vein compared with the tail vein during multiple blood sampling of mice. Lab Anim. 2010;44:25–29. 10.1258/la.2009.009017. 19535392 10.1258/la.2009.009017

